# A Novel In Vitro Host−Pathogen Model for *Felis catus* and *Sporothrix* Zoonotic Species Investigation

**DOI:** 10.1002/jobm.70077

**Published:** 2025-07-14

**Authors:** Gabriele Barros Mothé, Nathália Faria Reis, Emylli Dias Virginio, Miguel Angelo da Silva Medeiros, Adriany Lucas dos Santos, Júlia Andrade de Castro Rodrigues, Ricardo Luiz Dantas Machado, Gutemberg Gomes Alves, Nathália Curty de Andrade, Leila Maria Lopes‐Bezerra, Andréa Regina de Souza Baptista

**Affiliations:** ^1^ Center for Microorganisms' Investigation, Biomedical Institute Federal Fluminense University Niterói Rio de Janeiro Brazil; ^2^ Research Center for Cell Biology and Omics Rio de Janeiro State University Rio de Janeiro Brazil; ^3^ Castelo Branco University Rio de Janeiro Brazil; ^4^ Cell and Molecular Biology Department, Institute of Biology Fluminense Federal University Niterói Rio de Janeiro Brazil; ^5^ Center for Innovation, Entrepreneurship and Technology USP/IPEN/CIETEC São Paulo University São Paulo Brazil

**Keywords:** cat, host−pathogen interaction, immune response, phagocytosis, sporotrichosis

## Abstract

Hyperendemic zoonotic sporotrichosis, attributed to *Sporothrix brasiliensis*, presents a significant public health challenge in Brazil. Cats exhibit severe symptoms and high fungal loads, though their susceptibility is unclear. *Sporothrix schenckii* can also cause feline disease, primarily seen in Asia. This study is the first to report an in vitro model for examining cat immune cell responses to *S. brasiliensis* or *S. schenckii*. We investigated the phagocytic activity of blood cells (FMdP) from healthy domestic cats, challenged with yeast cells of *S. brasiliensis* and *S. schenckii*. The survival of these yeasts within cat phagocytes and their cytotoxic effect on host cells were monitored. Both fungal species developed and replicated within feline phagocytes while *S. brasiliensis* phagocytic index (PI) was higher (*p* < 0.0001). Interspecies analyses showed that *S. schenckii* required a higher multiplicity of infection to be more cytotoxic than *S. brasiliensis* (*p* ≤ 0.01). The present report brings relevant information to understand *S. brasiliensis* host adaptation and, ultimately, cat susceptibility to sporotrichosis. This pioneering study on the feline's innate immune response provides new insights for future complex studies such as those involving fungal ligand recognition by cat cell receptors.

**Abbreviations**
3R's: Replacement, Reduction, and Refinement
ALT: alanine aminotransferase
ANOVA: analysis of variance
ATCC: American Type Culture Collection
CAPES: Coordination for the Improvement of Higher Education Personnel
CEUA: Ethics Committee for the Use of Animals
CIM: Center for Microorganisms' Investigation
CNPq: National Council for Scientific and Technological Development
DMEM: Dulbecco Modification of Minimum Essential Media
FMdP: Feline Monocyte‐derived Phagocyte
GGT: gamma‐glutamyltransferase
GLU: glutamine
LDH: lactate dehydrogenase
MOI: Multiplicity of Infection
PBMC: peripheral blood mononuclear cells
PEN: penicillin
PI: phagocytic index
SD: standard deviation
STREP: streptomycin
TNF‐α: tumor necrosis factor
UFF: Federal Fluminense University
UFS: Universal Feline Sera
UFSin: inactivated Universal Feline Sera
YPD: yeast extract peptone dextrose

## 1 Introduction

There are two zoonotic species within the *Sporothrix* genus reported in the literature, with distinct geographical distributions, *Sporothrix brasiliensis* and *Sporothrix schenckii* [1, 2]. Animal‐transmitted sporotrichosis has seen a significant increase throughout Brazil, with the disease transitioning from isolated outbreaks to hyperendemic levels [1, 3]. In this context, domestic cats play a pivotal role as the primary animal species related to transmission and spreading sporotrichosis caused by *S. brasiliensis* [1, 3, 4]. In fact, cats are the most susceptible victims of this mycosis, often facing a poor prognosis due to the uncontrolled spread of the fungus [4, 5, 6].

Feline sporotrichosis is being reported for a long time in Asia but, in this case, the disease is caused by *Sporothrix schenckii* and had never reached the same clinical‐epidemiological impact [2]. Recently, *S. brasiliensis* crossed the Brazilian border and has been described in autochthonous cases in other Latin American countries, such as Argentina, Chile, and Paraguay [7, 8, 9]. In 2022, the first human cases of zoonotic sporotrichosis in Europe were reported, resulting from trauma by cats migrating from Brazil to the United Kingdom [10, 11].

Moreover, the therapeutic response in felines is complex due to multiple coexisting factors, such as the occurrence of relapsing and refractory cases [6, 12]. This suggests that, in addition to the virulence of the fungus, feline immunity may exert a relevant part on the poor ability to control *Sporothrix* and, therefore, disease severity. The host immune response to *Sporothrix* was investigated mainly in murine, human [13, 14, 15], and invertebrate models [16, 17]. Nonetheless, very little is known about the cats' immune response against *Sporothrix* spp [5, 18].

According to the damage‐response framework for fungal diseases, the symptoms and severity in the host are shaped not only by the virulence factors of the microorganism but also by the host's immune response [19]. In this context, the feline immune response may play a crucial role in their exceptional susceptibility to sporotrichosis. This study aimed to investigate, for the first time, the in vitro interaction between feline phagocytes and the two primary pathogens responsible for zoonotic sporotrichosis. To our knowledge, this is the first research to propose an in vitro protocol that explores the early immune events mediating the feline response to *Sporothrix* spp.

## 2 Materials and Methods

### 2.1 Ethical Aspects

This study was approved by the Ethics Committee for the Use of Animals (CEUA) of the Fluminense Federal University under protocol number 7561040518 on June 14, 2018.

### 2.2 Clinical Evaluation and Sample Collection

Blood cells were obtained from 40 mL blood bank bags, collected from domestic cats registered as donors at the Someve Veterinary Clinic, Rio de Janeiro, Brazil. These were included regardless of castration, sex, or breed, as long as they were adults and presumed healthy according to clinical and laboratory assessments. To this end, the veterinarian staff determined their general health condition, as well as hematological and biochemical tests (creatinine, urea, alanine aminotransferase [ALT], gamma‐glutamyltransferase [GGT], total protein and fractions). The snap test (IDEXX, Brazil) for feline immunodeficiency and leukemia viruses, and the anti‐SsCBF ELISA were also used to exclude previous FIV/FeLV and *Sporothrix* spp. infection [20], respectively.

### 2.3 Sera Processing, Isolation, and Preparation of Blood Cells

As described in item 2.2, adult healthy felines were included regardless of castration, sex, or breed. Fifteen milliliters of blood samples without anticoagulants were centrifuged at 700*g* for 10 min to separate the serum, later collected and divided into aliquots. A maximum of 2 mL of the remaining serum was successively placed in a 15 mL Falcon tube, on top of previous frozen sera (summing up to four distinct animals), adding to the pool named “Universal Feline Sera” (UFS), stored in a −80°C freezer. To guarantee the sterility of the experiments, UFS was previously filtered through a 0.22 μm pore filter (Corning, NY, USA).

Isolation of blood cells by density gradient after centrifugation was performed, as described by Lopes‐Bezerra et al. [21], with adaptations. Briefly, 20 mL of blood from feline blood bank bags with CPDA‐1 (JP indústria farmacêutica, S.A., SP, Brazil) was transferred to a 50 mL Falcon tube and diluted in 1x Hanks' solution (HBSS, Cultilab, SP, Brazil) in a 2:1 ratio. This content was transferred to another 50 mL Falcon tube containing Histopaque‐1077 (Sigma, Aldrich, USA) in a 2:1 ratio, centrifuged at 415*g* for 30 min at 20°C. Peripheral blood mononuclear cells (PBMCs) were collected and transferred to a new 50 mL Falcon tube and washed three times with 1x Hanks' balanced solution (HBSS, Cultilab, SP, Brazil) under successive centrifugations at 472*g*, 362*g*, and 266*g*, respectively, for 10 min. A final resuspension in DMEM (Dulbecco Modification of Minimum Essential Media, LGC Biotecnologia, SP, Brazil) was followed by cell counting in a Neubauer chamber and viability assessment by Trypan blue (Gibco, NY, USA) staining.

### 2.4 Microorganisms

American Type Culture Collection (ATCC) strain MYA 4823 (*S. brasiliensis*) and ATCC strain MYA 4820 (*S. schenckii)* were used as references. Both come from feline clinical isolates and had their virulence profile determined in a murine model, in addition to cell wall characterization [21, 22].

ATCCs were maintained on YPD agar medium (0.5% yeast extract, 1% peptone, 2% glucose, and 2% agar), and after growing at room temperature for 15 days, the stock cultures were kept at 4°C. The parasitic yeast phase was obtained after growing the conidia in YPD pH 7.8 medium at 37°C for 4−7 days, under orbital shaking [21]. Then, the yeasts were collected and filtered through a 0.40 μm pore filter (Easy streiner, Greiner) to remove hyphal fragments, and later washed twice in DMEM (LGC Biotecnologia, SP, Brazil). Finally, Trypan blue‐stained yeast viability and density were verified in a Neubauer chamber.

### 2.5 *Sporothrix*‐Feline Monocyte‐Derived Phagocyte (FMdP) Interaction Study

This protocol was developed from the previously published investigation on the human phagocyte‐*Sporothrix* interaction method [14], with adaptations. The cells were cultured in 24‐well plates (Kasvi, Curitiba, PR, Brazil) using 13 mm circular coverslips (Kasvi, Curitiba, PR, Brazil). The plates were stored at 37°C in an atmosphere of 5% CO_2_. The culture medium consisted of DMEM supplemented with 10% (v/v) UFS and 1% (v/v) antibiotics (Pen/Strep/Glut—10,000 units/mL penicillin; 10,000 μg/mL streptomycin; 29.2 mg/mL glutamine; Gibco, NY, USA).

Plates to be used in the phagocytosis assay were kept under incubation for 1 day (24 h) until exposure to *S. schenckii* or *S. brasiliensis*. To test variables and establish the Feline cells‐*Sporothrix* interaction model, the Multiplicity of Infection (MOI) of *Sporothrix*:FMdP was 1:1 and 3:1. In addition, different interaction times were tested (1, 4, 18, and 24 h) and, after each time point, culture supernatant was collected, used immediately or stored at −80°C for future analysis.

#### 2.5.1 UFS Versus Universal Feline Inactivated Sera

Another set of experiments was conducted under the above conditions (subitem 2.5), using inactivated Universal Feline Sera (UFSin) obtained by UFS heating at 56°C, for 30 min. UFSin was previously filtered through a 0.22 μm pore filter (Corning, NY, USA).

### 2.6 Phagocytosis Test and Phagocytic Index (PI)

After the distinct interaction times between Feline phagocytic cells and *Sporothrix* spp. yeasts, the coverslips were stained using the Romanovsky method (Newprov, Pinhais, PR, Brazil), and then mounted on glass slides for analysis under light microscopy (Nikon, ELWD, Japan) at following magnifications: ×50, ×100, ×400, and ×1000. The number of yeasts endocytosed as well as the free yeasts were counted. At least 50 phagocytes were counted in a minimum of 10 high‐power fields. The number of yeasts endocytosed per phagocyte was then determined (×100). Photomicrographs were obtained in the ×100 optical magnitude. This experiment was performed three times, each time in triplicate. Under distinct magnification, cell morphology and yeast filamentation were also qualitatively evaluated. The PI, defined as the percentage of phagocytic cells multiplied by the average number of ingested yeast per cell, was calculated according to Taborda and Casadevall [23].

### 2.7 Cytotoxicity Assay

Pathogen‐mediated cytotoxicity was measured after domestic feline PBMCs cultivation in 96‐well plates (Kasvi, Curitiba, PR, Brazil) for 7 days, later seeded with 0.5 × 10^5^ FMdPs/well, infected with *Sporothrix* yeast (*S. schenckii* or *S. brasiliensis*) at MOI 1:1 and 3:1, and arrested after 4 h of interaction. Cytotoxicity was determined by a lactate dehydrogenase (LDH) assay using the CytoTox 96 Non‐Radioactive Cytotoxicity Assay kit (Promega, Madrid, Spain) and following the manufacturer's instructions. The results were analyzed by obtaining the optical density using a multimode microplate reader (Beckman Coulter, USA). All the cytotoxicity experiments were carried out in triplicate. The negative controls were the conditioned cell culture media, not inoculated with the yeasts, while the positive controls contained cultured yeasts only.

### 2.8 Statistical Analysis

All experiments were performed triplicate. The resulting data was stored in Microsoft Excel (2020, Microsoft, USA), processed, and analyzed using GraphPad Prism version 6.0 and R version 4.2.3 software. The Shapiro–Wilk test was used to evaluate whether the obtained data set was normally distributed. One‐way analysis of variance (ANOVA) with Tukey's post hoc test was applied to the PI evaluation while Kruskal–Wallis test, followed by Dunn's post hoc test were used to analyze cytotoxicity. The significance level applied to the study was 5%.

## 3 Results

### 3.1 Phagocytosis Assay

**Figure 1:**
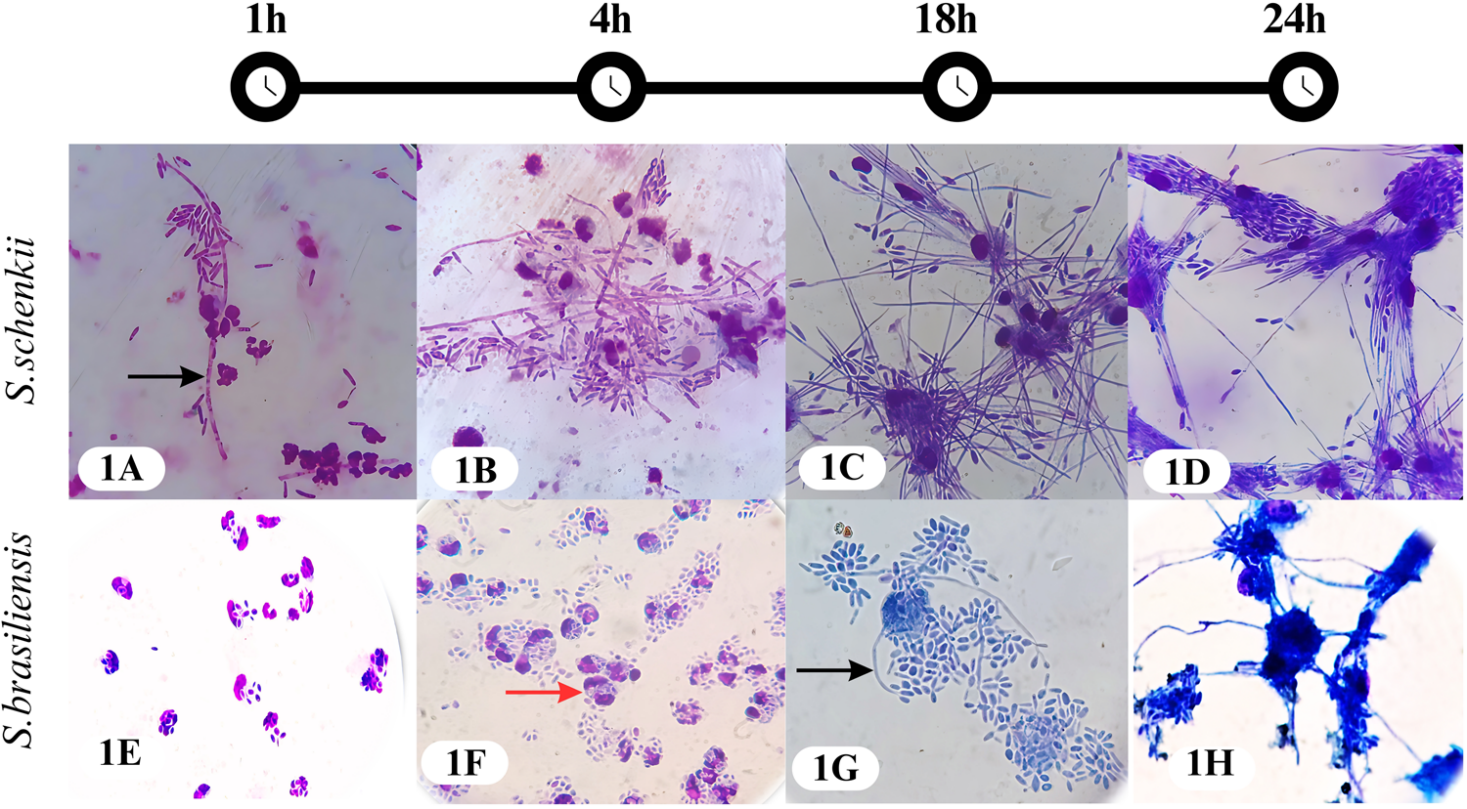
Interaction kinetics of *Sporothrix schenckii* or *Sporothrix brasiliensis* yeasts with feline phagocytes, during 1, 4, 18, and 24 h, stained with the Romanovsky method. Photomicrographs were obtained at a ×400 magnification. (A and G) Black arrows indicate early filamentation in *S. schenckii* and late filamentation in *S. brasiliensis*. (F) A red arrow indicates the interaction between *S. brasiliensis* and FMdP.

The ability of FMdP to recognize and internalize *S. schenckii* or *S. brasiliensis* yeasts was compared during interactions over four time periods: 1, 4, 18, and 24 h, as well as under different MOI ratios of *Sporothrix* to FMdP (1:1 and 3:1). Early exposure of yeast to phagocytes (1 h) proved effective (Figure 1), as extended exposure led to a significant increase in the number of internalized yeasts, particularly *S. brasiliensis* (*p* < 000.1), complicating counting due to yeast overlapping and/or clogging.

*S. schenckii* exhibited early filamentation within 1 h of interaction (Figure 1A), which progressively intensified over time (4−24 h). This phenomenon was observed both in internalized and non‐internalized yeasts, compromising the accurate assessment of phagocytic capacity (Figure 1C,D for *S. schenckii*). In contrast, *S. brasiliensis* lasted longer in its parasitic form, with slight filamentation, only becoming evident after 18 h of interaction (Figure 1G).

**Figure 2:**
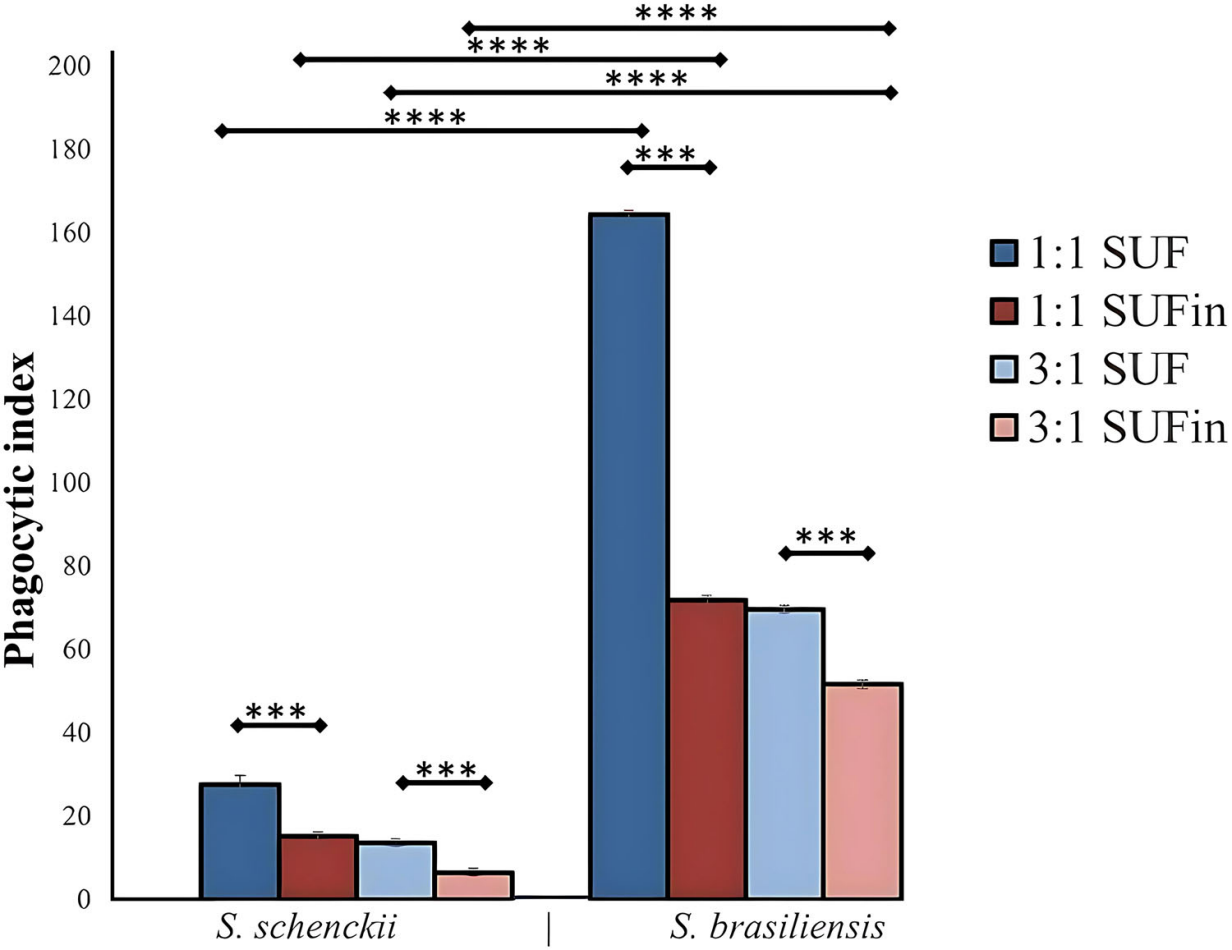
Phagocytic index of *Sporothrix schenckii* and *Sporothrix brasiliensis* by Feline Monocytes‐derived Phagocytes (FMdP) at MOI 1:1 or 3:1, with Universal Feline S,era (UFS) or inactivated Universal Feline Sera (UFSin). Results are the mean ± standard deviation (SD) of three experiments, in triplicate. Statistical analysis: ANOVA and Tukey's post hoc. Significant differences were estimated with *p* < 0.05 and were considered significant. *p ≤ 0.05, **p ≤ 0.01, ***p ≤ 0.001, and ****p ≤ 0.0001.

The PI obtained from FMdP interaction with *S. schenckii* and *S. brasiliensis* was significantly higher at MOI 1:1 (Figure 2; *p* < 0.001). The endocytosis of *Sporothrix* by FMdP, assessed in the presence of universal feline serum (UFS) and inactivated universal feline serum (UFSin), showed that serum inactivation reduced phagocytosis (Figure 2; *p* < 0.0001). This reduction was even more pronounced for *S. brasiliensis* compared to *S. schenckii* at MOI 1:1 versus MOI 3:1 (Figure 2; *p* < 0.0001; ANOVA test).

### 3.2 Cytotoxicity Assay

**Figure 3:**
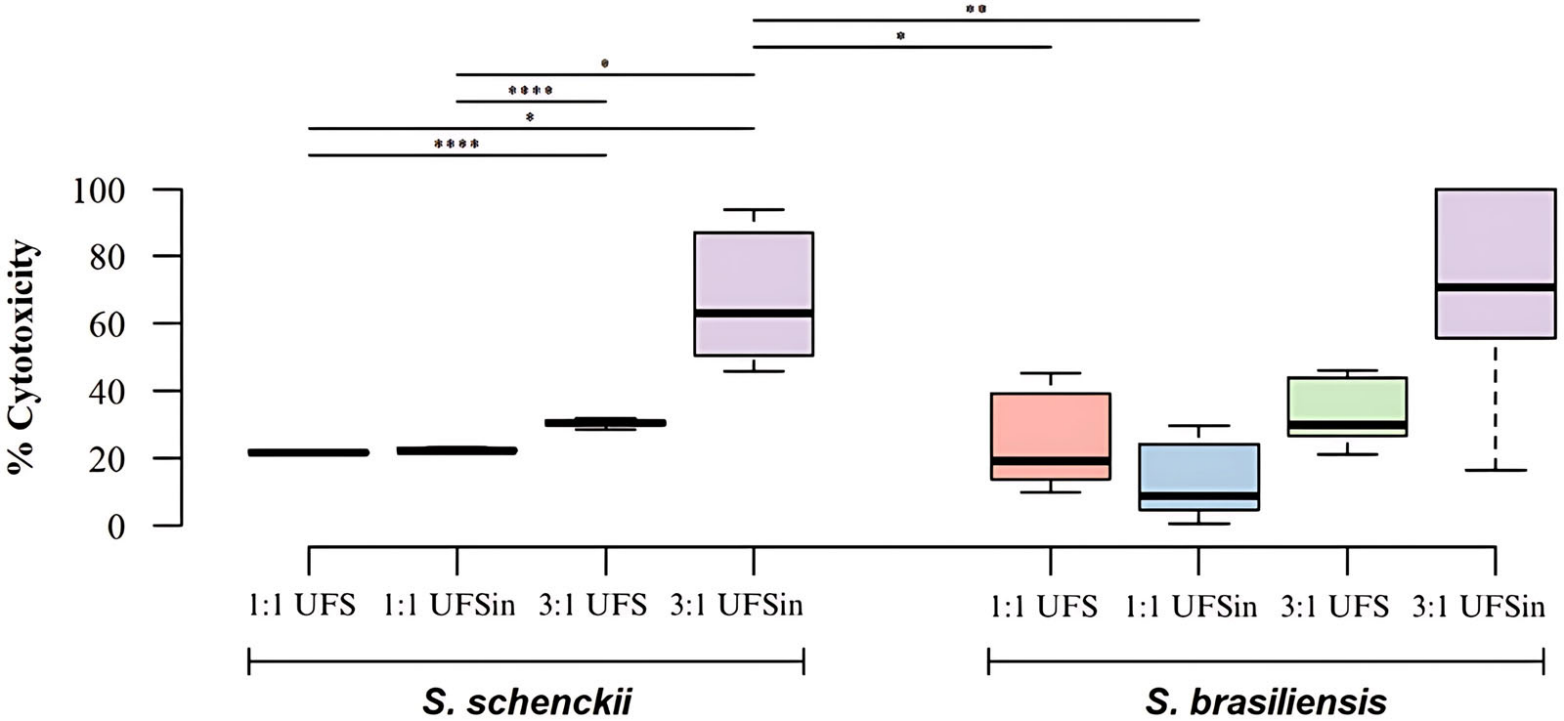
The cytotoxicity of *Sporothrix schenckii* and *Sporothrix brasiliensis* on FMdP cells under different Multiplicities of Infection (MOI) and culture supplemented media conditions (UFS/UFSin). Statistical analysis was performed using the Kruskal–Wallis test followed by Dunn's post hoc test. The results presented are the mean ± standard deviation (SD) from three independent experiments, each conducted in triplicate. Differences were considered significant if *p* < 0.05, with levels of significance indicated as follows: *p ≤ 0.05, **p ≤ 0.01, ***p ≤ 0.001, and ****p ≤ 0.0001.

The viability of feline cells was assessed following a 1 h exposure to *S. schenckii* or *S. brasiliensis*. Both pathogens exhibited cytotoxic effects on FMdP cells that had been cultured for 7 days. The cytotoxicity caused by *S. brasiliensis* ranged from 15% to 70%, with similar results between different conditions (MOI 1:1 or 3:1) or the use of UFS or UFSin (*p* > 0.05). *S. schenckii* showed cytotoxicity ranging from 20% to 60%, significantly higher at elevated MOI, regardless of serum inactivation: UFS (MOI 1:1 and 3:1; *p* < 0.0001) and UFSin (MOI 1:1 and 3:1; *p* < 0.05). Analyses revealed *S. schenckii* needed a higher MOI to be more cytotoxic to FMdP than *S. brasiliensis* in UFSin supplemented media (*p* ≤ 0.01) (Figure 3).

## 4 Discussion

Although several other hosts are susceptible to *Sporothrix* infections, domestic cats stand out as the species capable of directly transmitting the disease while develop its most severe forms [1, 5]. Unveiling the mechanisms involved in host−pathogen interactions may ultimately offer valuable insights for identifying future targets for disease treatment and prevention. Despite the efforts toward a better understanding of interactions between *Sporothrix* spp. and the feline immune system, much of the progress that has been performed by in vitro and in vivo experimental models is yet to be explored in this host. We conducted a novel investigation into the interactions between *Sporothrix* and feline immune cells, utilizing a modified protocol originally developed for studying human immune responses [14].

This work reports that *S. schenckii* and *S. brasiliensis* have the capability to recognize, survive, develop, and replicate within feline phagocytes, mirroring the typical phenomena observed during cytopathological examination of cat sporotrichoid lesions [24]. Notably, other fungal pathogens responsible for severe fungal diseases, such as *Histoplasma capsulatum* and *Cryptococcus neoformans*, are capable of withstanding the microbicidal activity of human phagocytes. This survival contributes to the persistence of chronic and latent infections [25, 26]. Likewise, *S. brasiliensis* possesses the capability to endure and replicate within human phagocytes [14].

Macrophages have been demonstrated to respond to opsonised *S. schenckii* conidia and yeast via various phagocytic receptors, resulting in a reduced pro‐inflammatory response and a decreased rate of ROS‐induced cell death, thereby enhancing pathogen survival [27]. In the present study, *S. schenckii* exhibited early and progressively increasing filamentation within 1 h, whereas *S. brasiliensis* remained in its parasitic form for a longer duration, only showing slight signs of filamentation after 18 h of interaction. The conversion to the yeast parasitic phase at temperatures above 37°C (thermotolerance) may be considered a virulence factor that benefits *S. brasiliensis*. Neves et al. [14] reported the germination of *S. brasiliensis* following an 18 h interaction within human macrophages. Routine feline sporotrichosis diagnostic cytopathology smears occasionally show *S. brasiliensis* hyphae and daisy‐like conidia arrangement (unpublished data). One might hypothesize that the alternation between morphotypes is crucial for *Sporothrix* adaptation to both the tissue microenvironment [28, 29] and the higher body temperatures of domestic cats and dogs (38−39°C). Additionally, for the yeast to spread from the cutaneous site of infection to other tissues or organs, it is advantageous for it to remain in its unicellular form, *Sporothrix* parasitic phase.

In light of human findings [14, 27], to ascertain whether thermolabile serum factors might affect the uptake of *S. schenckii* and *S. brasiliensis* yeast cells by FMdP, interaction assays were conducted utilizing a medium supplemented with heat‐inactivated feline sera. The uptake of yeasts by FMdP for these two fungal species was reduced but not entirely abolished by host serum inactivation, similar to the findings observed in human models [14].

Regardless of the culture protocol variables (MOI, interaction times, UFS, or UFSin), *S. brasiliensis* had a higher uptake by FMdP compared to *S. schenckii*. This finding aligns with previous murine models [13] and human in vitro experimental infections [14, 21]. Likewise, the feline defense cells mirror the recognition of *S. schenckii* and *S. brasiliensis* yeasts, as described for the human macrophages. The overall percentage of cytotoxicity induced by *S. brasiliensis* was similar to that observed with *S. schenkii*, amounting to 60%−70%. In heat‐inactivated supplemented media, comparative analyses indicated that *S. schenckii* required a higher MOI to demonstrate increased cytotoxicity relative to *S. brasiliensis*. Previously, the cytotoxic effects of *S. brasiliensis* were attributed to tumor necrosis factor (TNF‐α) secretion by human and murine macrophages [14, 30]. Further research involving cytokine quantification is required to ascertain if feline immune cells exhibit comparable microbicidal mechanisms.

One recognized limitation in extrapolating the FMdPs assay to human hosts is the unique physiological characteristics of each species. The first initial concerns pertain to technical limitations, including the reduced volume post‐blood collection and, consequently, the total number of cells obtained. For instance, commercial kits validated for species‐specific evaluation, such as those designed for cytokine assessment, are not always as readily available as those intended for human hosts. Also, the specific requirements imposed by each country's ethics committees and regulations concerning the use of animals in scientific research may delay the establishment of a standardized protocol.

Additionally, although there are limited studies on feline immunity [5, 18, 31], it is understood that significant differences exist in the recognition of pathogens and the subsequent cascades initiated during infectious processes. It is noteworthy that the immunological recognition of key components of the cell walls of pathogenic fungi infecting mammals remains largely unexplored [32]. Miranda et al. [31] and subsequently Souza et al. [33] described the histopathological characteristics of feline sporotrichotic lesions. They observed that, in contrast to humans, domestic cats have difficulty to develop suppurative granulomas, with significant quantities of yeast occupying macrophages within those lesions. These data suggest a hypothesis that there is a correlation between barely formed granulomas and the absence of epithelioid cells, coupled with uncontrolled proliferation of *Sporothrix* spp. yeast, leading to poor disease prognosis.

While our PBMC‐derived phagocyte in vitro model does not fully reproduce the in vivo complex tissue environment—such as granuloma formation, intricate cell−cell interactions, and localized cytokine networks—it nonetheless provides a valuable and controlled platform to dissect the early events of feline immune response to *Sporothrix* spp. This model enables us to pinpoint key cellular processes and pathogen strategies that may underlie the poor granulomatous response observed in feline tissues. As such, the insights gained here lay a foundation for future studies that may incorporate additional tissue‐specific variables or in vivo confirmation, bridging the gap between reductionists in vitro findings and the complexity of in situ feline sporotrichosis.

The findings indicate that *S. brasiliensis* has a higher PI and sustains the parasitic form longer than *S. schenckii*. Both species show significant cytotoxic effects on feline phagocytes. This suggests a possible mechanism for the higher severity observed in feline sporotrichosis. Our findings suggest potential mechanisms in pathogenesis and identify key targets for future control of this neglected zoonosis. This study paves the way for developing dynamic in vitro models to study cat pathogens. An enhanced protocol has the potential to considerably diminish the reliance on animals in pathogen−host interactions and drug testing research. This advancement would support the ethical principles of the 3R's: Replacement, Reduction, and Refinement.

## Author Contributions

**Gabriele Barros Mothé:** conceptualization, data curation, formal analysis, investigation, methodology, validation, writing – original draft. **Nathália Faria Reis:** validation, writing – original draft, writing – review and editing, methodology, investigation, visualization. **Emylli Dias Virginio:** methodology, formal analysis, investigation. **Miguel Angelo da Silva Medeiros:** investigation, methodology, validation. **Adriany Lucas dos Santos:** investigation, writing – review and editing, visualization. **Júlia Andrade Castro Rodrigues:** investigation, writing – review and editing, data curation. **Ricardo Luiz Dantas Machado:** methodology, writing – review and editing, resources. **Gutemberg Gomes Alves:** validation, writing – review and editing, resources, data curation. **Nathália Curty de Andrade:** conceptualization, methodology, formal analysis, investigation. **Leila Maria Lopes‐Bezerra:** conceptualization, formal analysis, writing – review and editing, resources, funding acquisition. **Andréa Regina de Souza Baptista:** conceptualization, writing – original draft, writing – review and editing, funding acquisition, supervision, project administration, resources.

## Conflicts of Interest

The authors declare no conflicts of interest.

## Data Availability

The data that support the findings of this study are available from the corresponding author upon reasonable request.
